# Identification and validation of a histone modification-related gene signature to predict the prognosis of multiple myeloma

**DOI:** 10.3389/fgene.2025.1613631

**Published:** 2025-08-28

**Authors:** Juan Lyu, Shanmei Lyu, Ying Qian, Yi Feng, Zhuan Zheng, Lihong Zhang

**Affiliations:** Department of Clinical Laboratory Center, Shaoxing People’s Hospital, Shaoxing, Zhejiang, China

**Keywords:** mm, prognosis, histone modification, gene signature, cell cycle

## Abstract

**Background:**

Multiple myeloma (MM) is an incurable plasma cell malignancy with high heterogeneity. Current staging systems, including the International Staging System (ISS) and Revised ISS (R-ISS), have limited prognostic accuracy. Given the role of histone modifications in MM progression, we developed a histone modification-related (HMR) prognostic model to improve MM risk stratification.

**Methods:**

Gene expression and mutation data were downloaded from the Gene Expression Omnibus database and the Cancer Genome Atlas. Prognostic HMR-related genes were identified through a combination of univariate Cox regression, least absolute shrinkage and selection operator Cox regression, and random survival forest analysis. The genes identified were then used to construct the HMR risk score model using multivariate Cox regression. The model was validated using Kaplan-Meier survival, time-dependent receiver operating characteristic curves analysis. A nomogram combining the HMR score with clinical features was developed. Functional enrichment, immune infiltration, somatic mutation, and drug sensitivity analysis were conducted to explore the biological relevance of the model.

**Results:**

Seven HMR genes with prognostic significance were identified. The HMR risk score stratified patients into high-risk and low-risk groups, with significant survival differences. The model demonstrated favorable predictive performance, and was shown to be an independent prognostic factor. The nomogram showed good calibration and discriminative ability, offering a practical tool for individual patient risk assessment. Functional analysis revealed that the HMR risk score is associated with dysregulated cell cycle progression, proliferation, and immunosuppression in MM, which may contribute to disease progression and drug resistance. Moreover, drug sensitivity analysis indicated potential associations between the HMR score and response to specific therapeutic agents, highlighting its potential role in guiding personalized treatment.

**Conclusion:**

We developed an HMR gene signature that has potential for prognostic prediction and may help guide personalized treatment strategies in MM.

## Introduction

Multiple myeloma (MM) is a malignant plasma cell disease that accounts for about 10% of all hematological cancers, and is the second most common hematologic cancer worldwide (Siegel et al., 2022; Zhuge et al., 2025). Over the last 30 years, MM’s worldwide incidence and mortality rates have more than doubled (Zhuge et al., 2025). The clinical manifestations include renal impairment, hypercalcemia, lytic bone lesions, and anemia (Kyle and Rajkumar, 2008). Despite the continuous emergence of novel therapeutic agents and due to the heterogeneity in pathogenesis, the prognosis for MM patients remains poor (van de Donk et al., 2021; Attal et al., 2017; Karimi et al., 2025). The widely used International Staging System (ISS) is based on albumin (ALB) and β2-microglobulin (B2M) (Girija Sivasankaran et al., 2025). However, it lacks genetic and molecular markers, limiting its accuracy in guiding individualized treatment and prognosis assessment (Hagen et al., 2022). To further improve the prognosis, it is crucial to identify and integrate additional prognostic biomarkers to refine risk stratification, improve outcome prediction, and optimize treatment selection.

Epigenetic modifications are reversible heritable changes in gene expression that do not alter the DNA sequence. Epigenetic variations are achieved through covalent chemical modifications of chromatin, including DNA and histone modifications, chromatin remodeling, and non-coding RNAs (Nagano and Fraser, 2009; Chervona and Costa, 2012; Lee and Kim, 2022). Among these, histone modifications, such as methylation, acetylation, phosphorylation, adenylylation, ubiquitination, and ADP-ribosylation occur at histone tails and play a crucial role in regulating gene expression (Kouzarides, 2007). These modifications regulate gene expression by altering chromatin structure or influencing transcription factor binding to gene promoters (Li et al., 2007). Histone modifications are closely associated with various biological processes, including gene expression regulation, cell differentiation and development, the cell cycle, tumorigenesis, immune cell infiltration, and cancer prognosis (Chervona and Costa, 2012; Ji et al., 2025; Sharma et al., 2010; Bi et al., 2020).

Histone modifications play a crucial role in regulating key gene expression and influencing disease progression in MM (Ismail et al., 2022). Abnormal expression of histone-modifying enzymes, such as histone deacetylases (HDACs) and histone methyltransferases (HMTs), disrupts transcriptional balance in MM cells, thereby affecting cell proliferation, apoptosis, and drug resistance. Targeting histone modifications has emerged as a promising therapeutic strategy, with HDAC inhibitors and EZH2 (Enhancer of Zeste Homolog 2) inhibitors showing significant potential in MM treatment (Nylund et al., 2021; Pu et al., 2024). Among the genetic abnormalities in MM, the t(4;14) translocation occurs in 15%–20% of patients, leading to FGFR3 (fibroblast growth factor receptor 3) and MMSET (multiple myeloma SET domain) overexpression. MMSET, a member of the NSD (Nuclear receptor-binding SET domain) family, possesses a SET domain responsible for histone methylation. Overexpression of MMSET in MM cells leads to globally elevated H3K36 dimethylation, accompanied by a significant reduction in H3K27 methylation. Additionally, MMSET binds to the IRF4 (interferon regulatory factor 4) promoter and regulates its expression, affecting cell growth, adhesion, and chromatin accessibility (Xie et al., 2015; Martinez-Garcia et al., 2011). Polycomb repressive complex 2 (PRC2) maintains the silent state of target genes such as HOX genes through trimethylation of H3K27, contributing to development and differentiation as well as tumorigenesis (Margueron and Reinberg, 2011). EZH2 is a core component of PRC2 that confers the HMT activity (Chase and Cross, 2011). Overexpression of EZH2 is linked to MM progression and poor prognosis (Pawlyn et al., 2017). EZH2 inhibitors reduce global H3K27me3 level and trigger apoptosis in MM cells (Hernando et al., 2016). HDAC inhibitors also impair MM cell growth and survival. Preclinical studies have demonstrated that HDAC inhibitors trigger apoptosis, as well as induce cell cycle arrest, in MM cells (Maiso et al., 2006).

In this study, we developed a histone modification-related (HMR) prognostic model comprising of seven genes to predict clinical outcomes in MM. Our findings not only elucidate the prognostic value of HMR genes but also establish a foundation for future research on targeted therapies strategies in MM.

## Materials and methods

### Data collection

Gene expression and clinical data of GSE24080, GSE136337, GSE136324, and GSE2658 were obtained from the Gene Expression Omnibus (GEO) database (http://www.ncbi.nlm.nih.gov/geo/). The data were preprocessed with normalization using the R package “limma”. The RNA sequence data and related somatic mutation data were acquired from the MMRF-CoMMpass project on the Genomic Data Commons Data Portal (https://portal.gdc.cancer.gov/). Patients with complete survival data and an overall survival time greater than 1 month were included in this study. Patients who did not meet these criteria or had incomplete clinical data were excluded from the analysis.

Dataset GSE24080 was used as the training cohort for model construction, while GSE136337, GSE2658, and MMRF-CoMMpass were used as validation cohorts. The MMRF-CoMMpass dataset provided somatic mutation data. GSE136324, which contains RNA extracted from the whole bone marrow (WBM) of MM patients, was used for immune infiltration analysis. Detailed information on all datasets used in this study is provided in Supplementary Table S1.

This study strictly adheres to data usage regulations and conducts secondary analysis based on publicly available data, thus requiring no additional ethical approval.

### Construction and validation of a HMR prognostic risk model

HMR genes were extracted from the “GOMF_HISTONE_MODIFYING_ACTIVITY” gene set in the Gene Set Enrichment Analysis (GSEA) database (http://www.gsea-msigdb.org/gsea/msigdb). After intersecting these genes with those detected in the GSE24080, GSE136337, GSE136324, GSE2658, and MMRF-CoMMpass, a total of 173 genes were selected for further analysis.

The GSE24080 dataset was used as the training cohort to create the HMR prognostic risk model. Univariate Cox regression was performed to identify genes related to prognosis (p value <0.01 and false discovery rates (FDR) < 0.05). The candidate genes identified were then subjected to two complementary feature selection methods to enhance robustness. First, Least Absolute Shrinkage and Selection Operator (LASSO) Cox regression was performed using the “glmnet” R package (v 4.1-8) to minimize overfitting and select features with non-zero coefficients. Ten-fold cross-validation was conducted to determine the optimal lambda value, resulting in the selection of 13 genes. Second, Random Survival Forest (RSF) analysis was carried out using the randomForestSRC (v 3.3.3) R package with 10 trees (ntree = 10) and one split per node (nsplit = 1). Variable importance was evaluated using the minimal depth method, and the top-ranked genes were retained. The intersection of genes identified by both LASSO and RSF methods yield seven genes: SUZ12, KAT2A, AURKA, BUB1, UTY, SUV39H2, and PCGF5. Pairwise Spearman correlation analysis was performed among the seven candidate genes. To further evaluate multicollinearity, the variance inflation factor (VIF) was calculated for each gene.

Subsequently, these genes were included in a multivariate Cox proportional hazards regression model to construct the final prognostic signature. A prognostic risk score was calculated for each patient as a linear combination of expression levels weighted by multivariate Cox coefficients: HMR score $=\sum_{i=1}^{n}\beta_{i}\times {\mathrm{Exp}}_{i}$ , where $\beta_{i}$ is the coefficient of gene i, and ${\mathrm{Exp}}_{i}$ denotes its normalized expression level. The formula was applied using the “predict” function in the “survival” package (v 3.8.3), with coefficients derived from the training cohort.

The optimal cutoff value for stratifying patients into high-risk and low-risk groups was determined using survminer R package (v 0.5.0), which identifies the threshold that best separates survival outcomes based on the log-rank test. To ensure comparability across datasets, the quantile corresponding to the optimal cutoff in the training cohort was calculated and applied to the validation cohorts to define high-risk and low-risk groups.

Finally, the prognostic value of the HMR risk score was evaluated using Kaplan–Meier (KM) survival analysis and time-dependent receiver operating characteristic (ROC) curves, using “survminer”, “survival”, and “timeROC” R packages.

### Establishment of predictive nomogram

Univariate and multivariate Cox regression analyses were performed on the HMR risk score and clinical characteristics to identify independent prognostic predictors. Variables with a p-value <0.05 were selected for further analysis. A combined model was constructed using the “rms” and “regplot” R packages to generate a nomogram. The predictive performance of the model was evaluated using calibration curves, the concordance index (C-index), and time-dependent ROC curves (using the “timeROC” package, v 0.4). The clinical benefit of the model was further assessed by decision curve analysis (DCA) using the “ggDCA” package (v 1.2).

Furthermore, the Wilcoxon rank-sum test was used to evaluate differences in HMR risk score across various clinical subgroups of MM patients.

### Differential expression genes (DEGs) analysis and pathway enrichment analysis

For GSE24080, DEGs between high-risk and low-risk groups were identified using the “limma” package (v3.62.2), with thresholds of |logFC| > 0.58 and adjusted p-value <0.05. For the MMRF-CoMMpass cohort, DEGs was conducted using the “DESeq2” package (v1.46.0), applying a threshold of |log2FoldChange | > 1 and adjusted p-value <0.05. The identified DEGs were then subjected to Gene Ontology (GO) and Kyoto Encyclopedia of Genes and Genomes (KEGG) pathway analyses using the “clusterProfiler” package (v4.14.4). To further validate the biological relevance, Gene Set Enrichment Analysis (GSEA) was performed using the “fgsea” package (v1.32.2), based on the preranked gene list ordered by the signal-to-noise ratio derived from differential expression between high-risk and low-risk groups.

### Evaluation of immune infiltration populations

We used a modified method described by Danziger et al. (2020) to calculate the immune cell infiltration levels in the bone marrow. Briefly, the whole bone marrow expression matrix was deconvoluted to calculate the fraction of 27 different cell types in WBM, including four types of plasma B cells. Then, the tumor immune microenvironment specific gene expression matrix was calculated according to the method described by Li et al. (2024).

### Evaluation of somatic mutations

Samples from the MMRF-CoMMpass project were divided into high-risk and low-risk groups as described above. The distribution of gene mutations across different groups was then visualized using the “maftools” R package (v 2.22.0).

### Drug sensitivity prediction

Drug sensitivity differences between the low-risk and high-risk groups were predicted using the “pRRophetic” R package (v 0.5) (Geeleher et al., 2014a). The dataset within the “pRRophetic” package is derived from the “cgp 2016” initiative, encompassing gene expression matrices and drug treatment information (Geeleher et al., 2014b). Wilcoxon test was used to identify drugs exhibiting different sensitivity between groups (p < 0.05).

### Statistical analysis

Statistical analysis was performed with R software 4.4.2. Continuous variables were compared using the Student’s t-test or Wilcoxon rank-sum test, depending on the normality test. Categorical variables were analyzed using the Chi-square test or Fisher’s exact test. Prognostic gene selection and model construction were performed using univariate and multivariate Cox regression, LASSO Cox regression, and RSF analysis. Survival differences were assessed by KM survival analysis and log-rank tests. Model performance was evaluated using time-dependent ROC curves and C-index. Clinical usefulness was assessed by DCA. Correlation analyses were performed using Spearman’s rank correlation test. A two-sided p-value <0.05 was considered statistically significant.

## Results

### Patient selection and statistics

**FIGURE 1:**
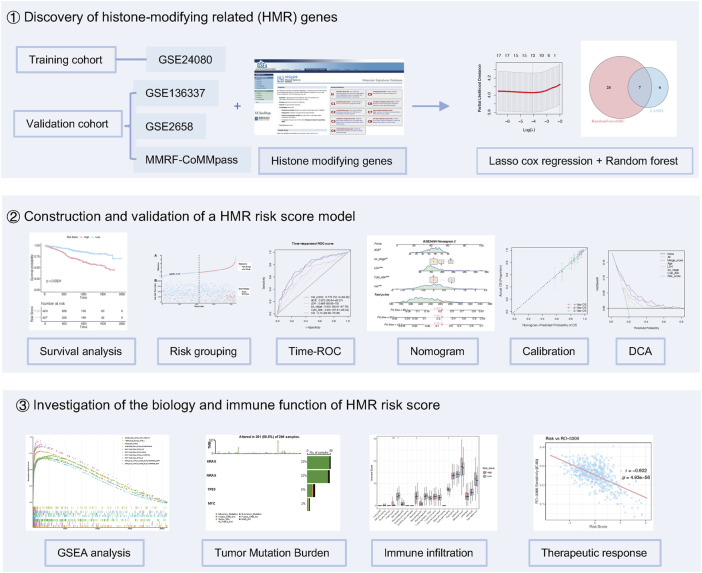
Study design for HMR risk model development.

This study included a total of 2790 MM patients from five datasets, with statistical details provided in Supplementary Table S1. The GSE24080 dataset (n = 556) was used as the training cohort to construct a HMR prognostic risk model. The GSE136337 (n = 424), GSE2658 (n = 555), and MMRF-CoMMpass project (n = 853) datasets were selected as validation cohorts. Somatic mutation data were extracted from the MMRF-CoMMpass project dataset (n = 804). The overall design and workflow of this study are illustrated in (Figure 1). Additionally, the GSE136324 dataset (n = 402) was used for immune infiltration analysis.

### Development of an HMR prognostic risk model

**FIGURE 2:**
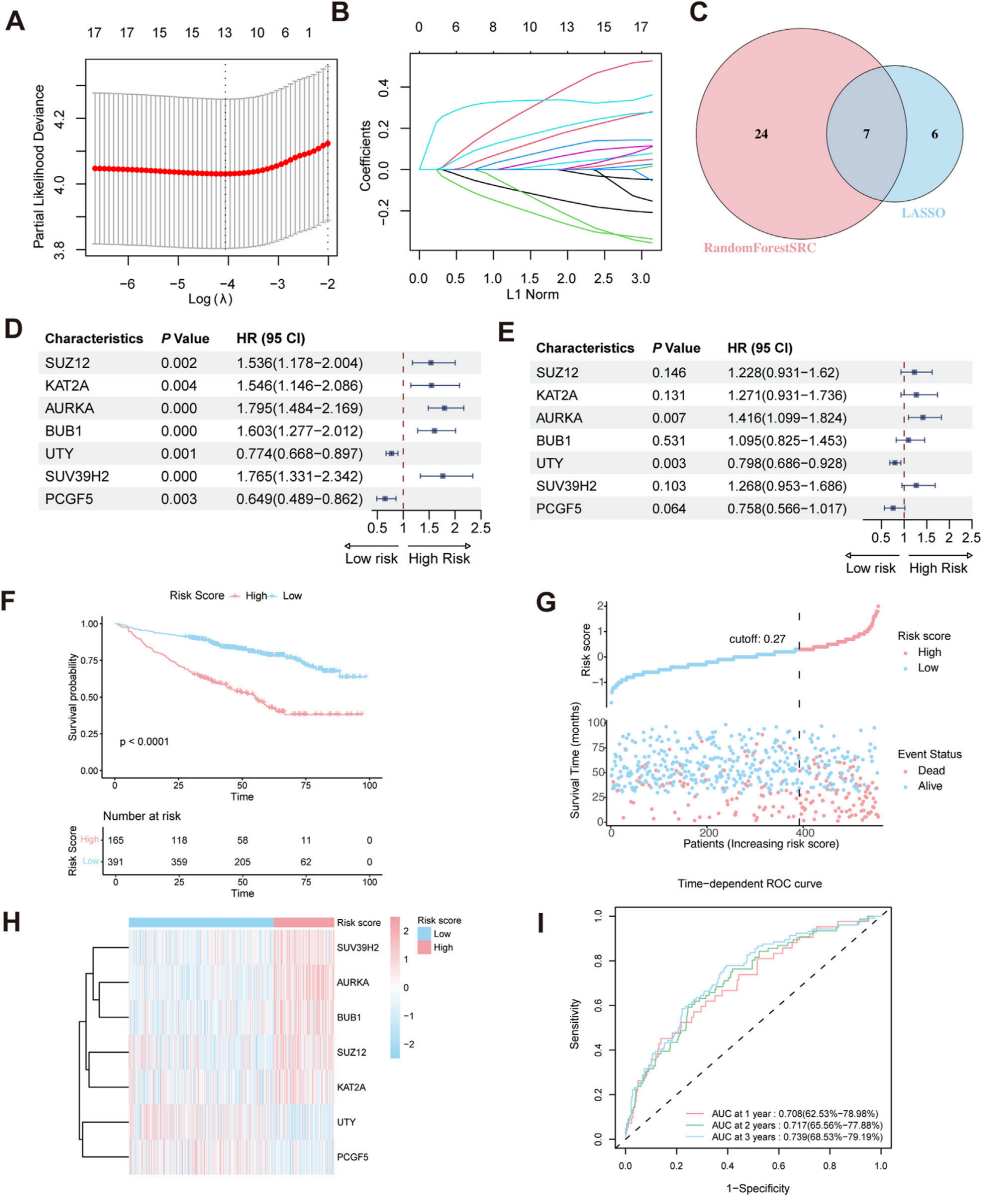
Construction of HRM prognostic risk model. **(A,B)** LASSO Cox regression analysis for variable selection. **(C)** Venn diagram shows the intersection of genes identified by LASSO regression and Random Forest analysis. **(D)** Forest plot of univariate Cox regression analysis in the GSE24080 training cohort. **(E)** Forest plot of multivariate Cox regression analysis in the training cohort. **(F)** Kaplan–Meier survival curve of high-risk and low-risk in the training cohort. **(G)** The distribution of risk scores and survival status of MM in the training cohort. **(H)** Heatmap of the HRM related genes in the training cohort. **(I)** Time-dependent ROC analysis in the training cohort.

To construct a HMR risk score, we identified seven HMR genes in the GSE24080 training cohort through univariate Cox regression, LASSO Cox regression analysis, and random forest analysis, as shown in (Figures 2A–C). Univariate Cox regression showed that SUZ12, KAT2A, AURKA, BUB1, and SUV39H2 were associated with poor prognosis (HR > 1, p < 0.05), indicating their potential role as high-risk genes. In contrast, UTY and PCGF5 were associated with favorable prognosis (HR < 1, p < 0.05), suggesting a protective effect (Figure 2D). Consistently, KM survival analysis based on median expression level further confirmed that high expression of SUZ12, KAT2A, AURKA, BUB1, and SUV39H2 correlated with worse prognosis, while high expression of UTY and PCGF5 suggested better prognosis (Supplementary Figure S1A–G).

Prior to model construction, pairwise correlations among the seven candidate genes were assessed using Spearman’s rank correlation analysis. The correlation coefficients ranged from −0.17 to 0.51 (Supplementary Figure S2A), indicating no strong collinearity. To further evaluate multicollinearity, the variance inflation factor (VIF) was calculated for each gene, all of which were <5 (Supplementary Figure S2B), confirming the absence of significant multicollinearity.

Multivariate Cox regression revealed that AURKA and UTY are independent prognostic risk factors in MM patients (Figure 2E). Based on the regression coefficient calculated by Multivariate Cox regression (Supplementary Figure S2C), the HMR risk score was calculated using the following formula: HMR risk score = (0.2052429×SUZ12 expression) +(0.2399438×KAT2A expression) +(0.3476679×AURKA expression) +(0.0905674×BUB1 expression) −(0.2257278×UTY expression) +(0.2372008×SUV39H2 expression) −(0.2765753×PCGF5 expression). Using this formula, we calculated the HMR risk score for each individual in both the training and validation cohorts. Subsequently, patients in the training dataset were stratified into high-risk and low-risk groups based on the optimal cutoff value. For the validation dataset, the corresponding quantile derived from the training dataset cutoff was used to define the risk groups.

### Validation of the HMR prognostic model

In the training dataset, KM survival analysis revealed that patients in the high-risk group had worse overall survival (Figure 2F). Risk distribution plot showed a higher proportion of deceased patients and shorter survival time in the high-risk group, further indicating a poorer prognosis (Figure 2G). The gene expression heatmap showed that high-risk genes (SUZ12, KAT2A, AURKA, BUB1, and SUV39H2) were upregulated in the high-risk group. Conversely, low-risk genes (UTY and PCGF5) exhibited higher expression in the low-risk group (Figure 2H). To evaluate the predictive performance of the HMR risk score model, we conducted a time-dependent ROC analysis. The area under the curve (AUC) at 1-year, 2-year, and 3-year intervals was 0.708 (95% CI: 62.5%–78.98%), 0.717 (95% CI: 65.56%–77.88%), and 0.739 (95% CI: 68.53%–79.19%), respectively (Figure 2I).

**FIGURE 3:**
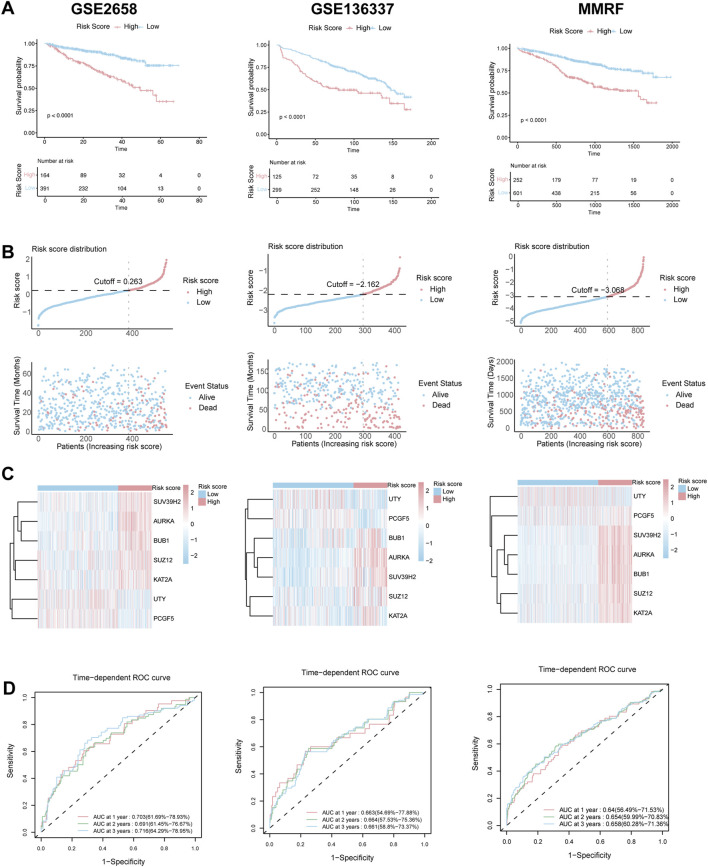
Validation of HRM prognostic risk model. **(A)** Kaplan–Meier survival curve of high-risk and low-risk in the GSE2658, GSE136337 and MMRF validation cohort. **(B)** The distribution of risk scores and survival status of MM in the GSE2658, GSE136337 and MMRF validation cohort. **(C)** Heatmap of the HRM related genes in the GSE2658, GSE136337 and MMRF validation cohort. **(D)** Time-dependent ROC analysis in the GSE2658, GSE136337 and MMRF cohort.

These findings were further validated in three independent validation cohorts (GSE2658, GSE136337, and MMRF-CoMMpass). Consistent with the training set, high-risk patients in these datasets had significantly worse survival outcomes, as shown by KM curves (Figure 3A), and exhibited similar patterns in risk distribution (Figure 3B), gene expression (Figure 3C), and time-dependent ROC performance (Figure 3D). Collectively, these results support the robustness of the HMR prognostic model across multiple datasets.

### HMR risk score is an independent prognostic factor in MM

To explore the relationship between the HMR risk score and clinical characteristics, we divided the patients in the GSE24080 training cohort into different subgroups based on clinical variables. The results of the Wilcoxon rank sum test showed that the HMR risk score was significantly elevated in female patients (P < 0.001), in those with higher levels of lactate dehydrogenase (LDH) (P < 0.001), beta-2-microglobulin (B2M) (P < 0.001), ISS stage (P < 0.001), those with cytogenetic abnormalities (Cyto Abn) (P < 0.001), and those with lower levels of albumin (ALB) (P < 0.05) (Supplementary Figure S1H–N). These results indicated that the HMR risk score correlated with adverse clinical parameters in MM.

Consistently, when comparing clinical characteristics between the high-risk and low-risk groups stratified by the HMR score, the high-risk group showed a significantly higher frequency of these unfavorable features (Supplementary Table S2), further confirming the close association between the HMR risk score and aggressive disease phenotypes in MM.

Next, Univariate and multivariate Cox regression analyses were performed to identify survival associated variables, including clinical parameters such as age, gender, ISS stage, LDH, Cyto Abn and HMR risk score in both training and validation cohort (Supplementary Figure S3). Notably, the HMR score demonstrated a p-value of <0.001 in both univariate and multivariate cox analyses across all cohorts, highlighting its potential as a robust and independent prognostic factor for MM patients.

### Nomogram construction for the clinical application of the HMR risk model

**FIGURE 4:**
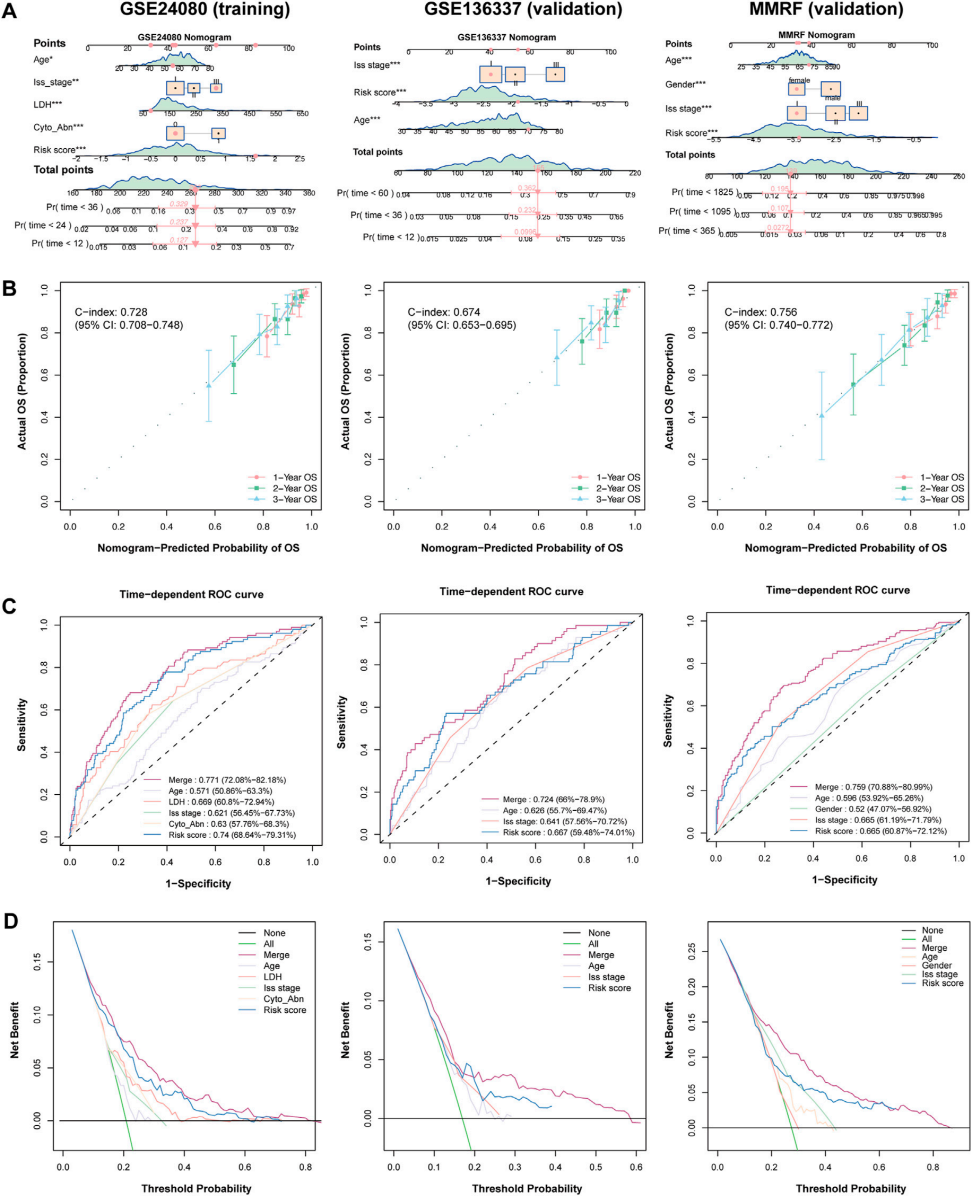
Establish and validation of predictive nomogram to evaluate clinical application. **(A)** Combined nomogram based on HRM risk score and clinical features in training (GSE24080) and validation (GSE136337 and MMRF) cohorts. **(B)** Calibration plot of nomogram plot in training (GSE24080) and validation (GSE136337 and MMRF) cohorts. **(C)** Time-dependent ROC curve in training (GSE24080) and validation (GSE136337 and MMRF) cohorts. **(D)** DCA curve in training (GSE24080) and validation (GSE136337 and MMRF) cohorts.

We further constructed a nomogram model that integrates clinical features and the HMR risk score to provide a quantitative tool for survival prediction (Figure 4A). Notably, the HMR risk score played a major role in the nomogram model, reflecting its strong prognostic value.

The nomogram demonstrated strong predictive performance in both the training and validation cohorts, with C-index value of 0.728 (95% CI: 0.708-0.748) in the training cohort, 0.674 (95% CI: 0.653-0.695) in the GSE136337 validation cohort and 0.756 (95% CI: 0.740-0.772) in MMRF-CoMMpass validation cohort, indicating good discriminative ability (Figure 4B). Calibration curves showed that the predicted survival probabilities at 1-year, 2-year, and 3-year survival were highly consistent with the actual survival rates in both cohorts, further confirming the reliability of the model (Figure 4B).

In the 3-year AUC analysis, the nomogram achieved an AUC of 0.772 in the trainig cohort. The HMR risk score achieved an AUC of 0.740, outperforming LDH (0.669) and ISS stage (0.621), emphasizing its superior prognostic relevance. Similarly, the nomogram achieved an AUC of 0.724 and 0.759 in GSE136337 and MMRF-CoMMpas validation cohort respectively (Figure 4C). Furthermore, Decision curve analysis (DCA) demonstrated that the nomogram provided the highest net benefit across different decision thresholds, with the HMR risk score ranking second and consistently outperforming other clinical variables in both training and validation datasets (Figure 4D).

Together, these findings indicate that the nomogram is a robust and reliable tool for individualized survival prediction in MM patients across both the training and validation cohorts. Notably, the HMR risk score showed superior prognostic performance compared to conventional clinical variables and contributed the most to the nomogram model, underscoring its pivotal role in risk stratification.

### GSEA enrichment analysis based on HMR risk score

**FIGURE 5:**
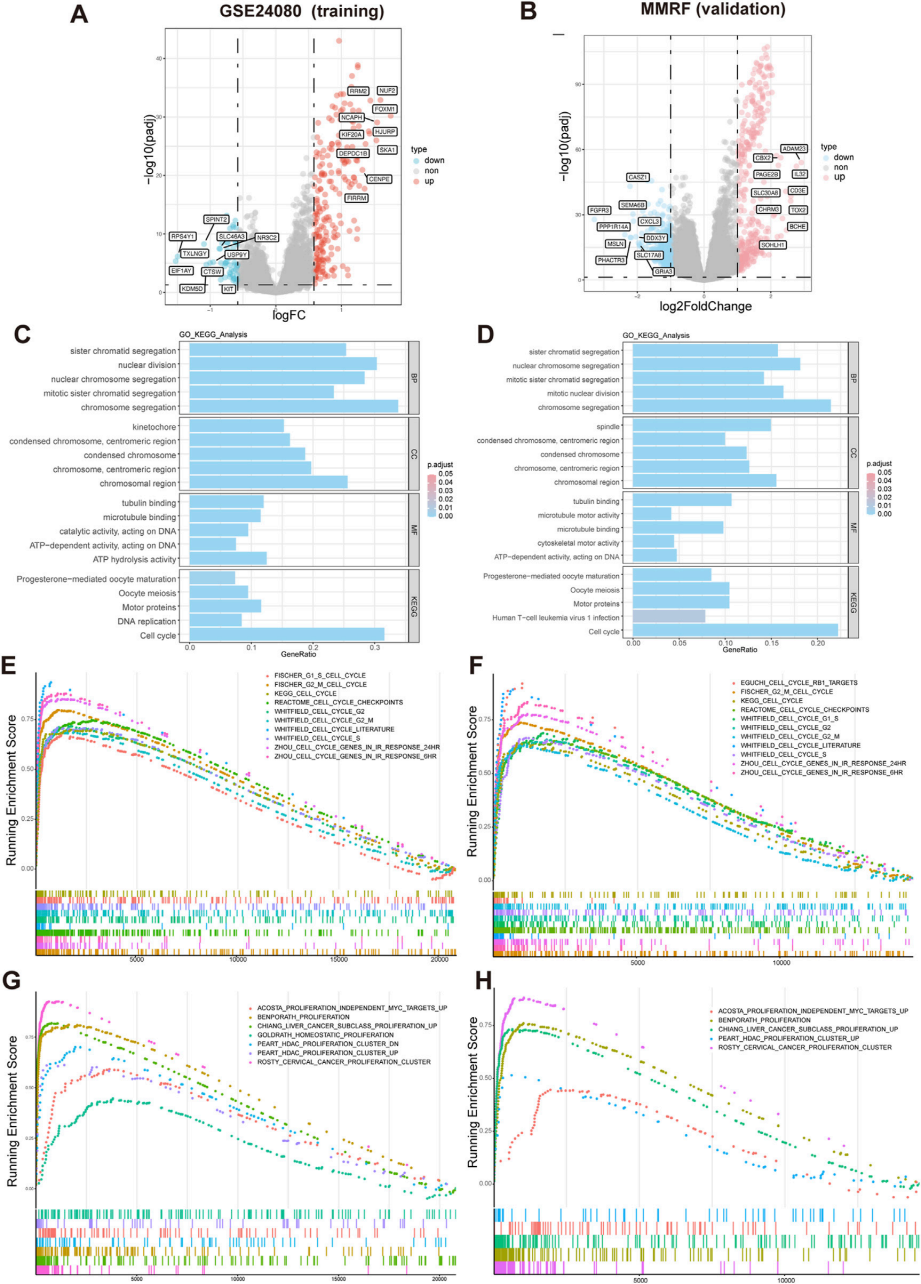
Differential expression and functional enrichment analysis based on HMR risk groups. **(A,B)** Volcano plot analysis of DEGs associated with HMR risk score in GSE24080 and MMRF datasets. **(C,D)** Gene Ontology (GO) and KEGG pathway enrichment analysis of DEGs. GSEA enrichment of DEGs associated with HMR risk score revealed a significant enrichment of gene set related to cell cycle **(E,F)** and proliferation **(G,H)** in the high-risk group.

Differential expression analysis identified 269 DEGs (|logFC| > 0.58, adjusted p-value <0.05), including 216 upregulated and 53 downregulated genes in GSE24080 dataset, and 663 DEGs (|log2FoldChange| > 1, adjusted p-value <0.05), including 359 upregulated and 304 downregulated genes in MMRF-CoMMpass dataset. Volcano plot visualization showed a distinct separation between upregulated and downregulated genes (Figures 5A,B). Gene Ontology (GO) analysis revealed that the significantly enriched biological processes (BP) were mainly involved in chromosome segregation and nuclear division. In the cellular component (CC) category, spindle and chromosomal region were predominantly enriched. The molecular function (MF) category was mainly associated with tubulin binding and microtubule binding. KEGG pathway analysis identified significant enrichment in cell cycle (Figure 5C,D). Consistently, GSEA demonstrated that gene sets related to cell cycle and proliferation were significantly enriched in the high-risk group (Figures 5E–H).

### Association between HMR risk score and tumor mutation burden (TMB)

**FIGURE 6:**
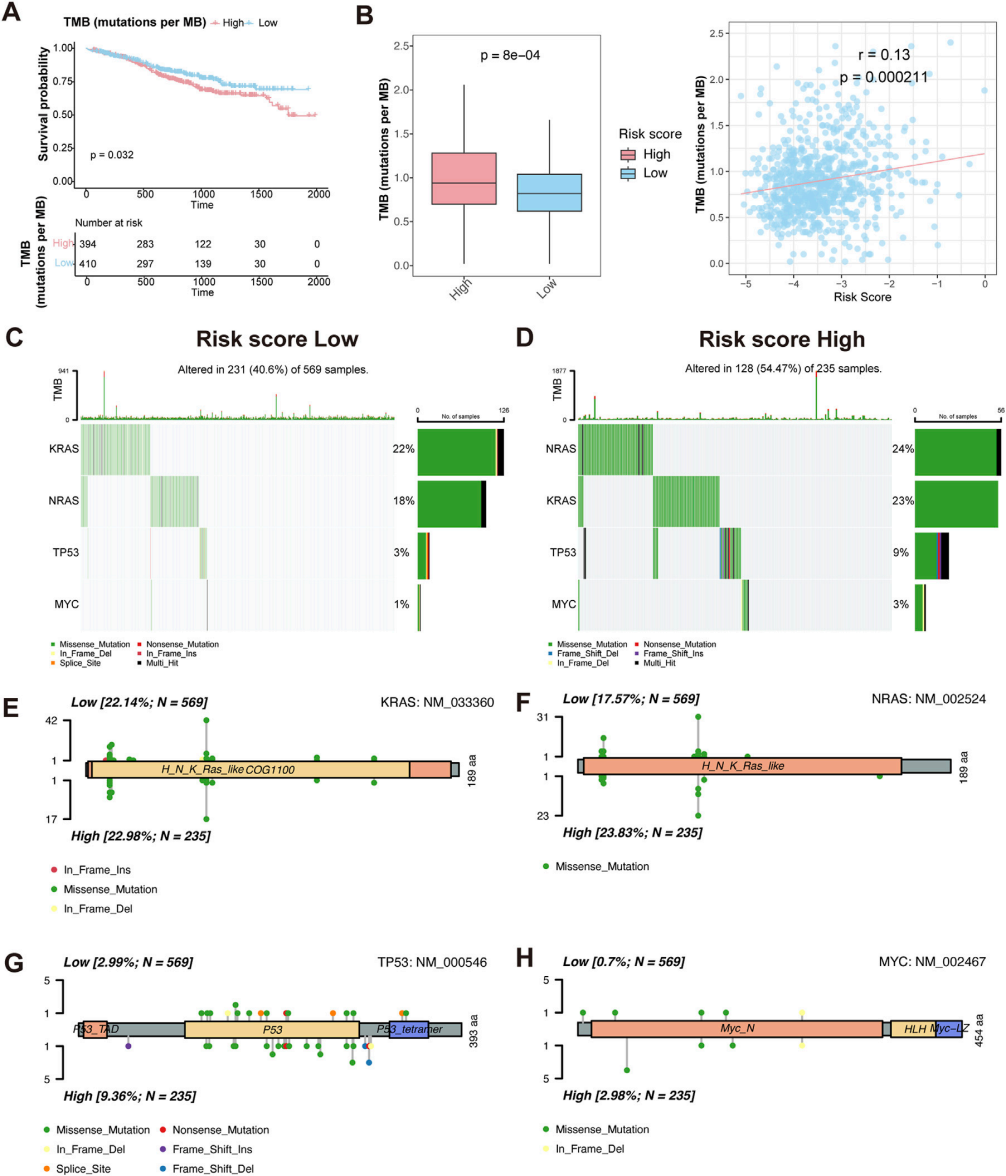
Characteristics of somatic mutation between HMR high-risk and low-risk groups. **(A)** Kaplan-Meier survival curves comparing overall survival between high and low TMB groups. **(B)** Boxplot comparing TMB levels between HMR high-risk and low-risk groups (left). Scatter plot and correlation analysis demonstrating a positive correlation between TMB and risk score (right). **(C,D)** Oncoplot visualizing mutation landscape of prognostic genes (KRAS, NRAS, TP53, and MYC) across risk groups. **(E–H)** Lollipop plots illustrating the distribution and frequency of mutations in KRAS, NRAS, TP53, and MYC in HMR high-risk and low-risk groups.

To investigate the relationship between TMB and prognosis in MM, we first performed KM survival analysis. The results showed that patients with high TMB had a worse overall survival compared to those with low TMB (P = 0.032) (Figure 6A), suggesting that TMB is an adverse prognostic factor.

We then assessed TMB distribution between HMR risk groups. The high-risk group exhibited a significantly higher TMB than the low-risk group (P = 7.6e-05), and a positive correlation was observed between TMB and HMR risk score (R = 0.13, P = 0.00021) (Figure 6B), indicating that genomic instability increases with higher HMR risk scores.

To further characterize genetic alterations in high-risk patients, we analyzed somatic mutations in key MM prognostic genes KRAS, NRAS, TP53, and MYC. The oncoplot and lollipop plot showed higher mutation frequencies of KRAS, NRAS, and TP53 in the high-risk group, particularly for TP53, indicating its key role in MM progression (Figures 6C–H).

### Correlation between HMR risk score and immune infiltration in bone marrow

**FIGURE 7:**
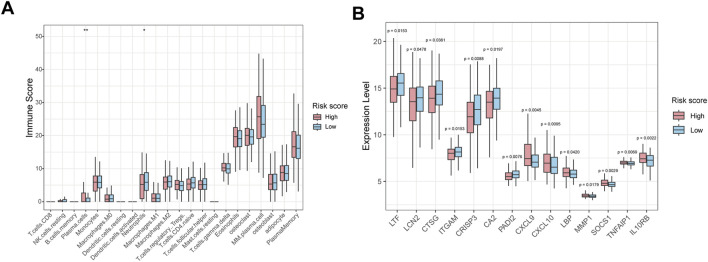
Immune infiltration and immune-related gene expression analysis between high-risk and low-risk groups. **(A)** Box plot showing the relative abundance of immune cell types in HMR high-risk and low-risk groups. **(B)** Box plots show immune-related gene expression levels in HMR high-risk and low-risk groups.

The immune infiltration analysis revealed that the abundance of neutrophils was significantly decreased in the high-risk group than in low-risk group (Figure 7A). Consistently, gene expression analysis showed that neutrophil-associated genes, including LTF, LCN2, CTSG, ITGAM, CRISP3, CA2 and PADI2 were upregulated in the low-risk group, while immune response and inflammation-related genes such as CXCL9, CXCL10, LBP, MMP1, SOCS1, TNFAIP1, and IL10RB were upregulated in high-risk group (Figure 7B).

### Prediction of chemotherapy sensitivity based on the HMR risk model

**FIGURE 8:**
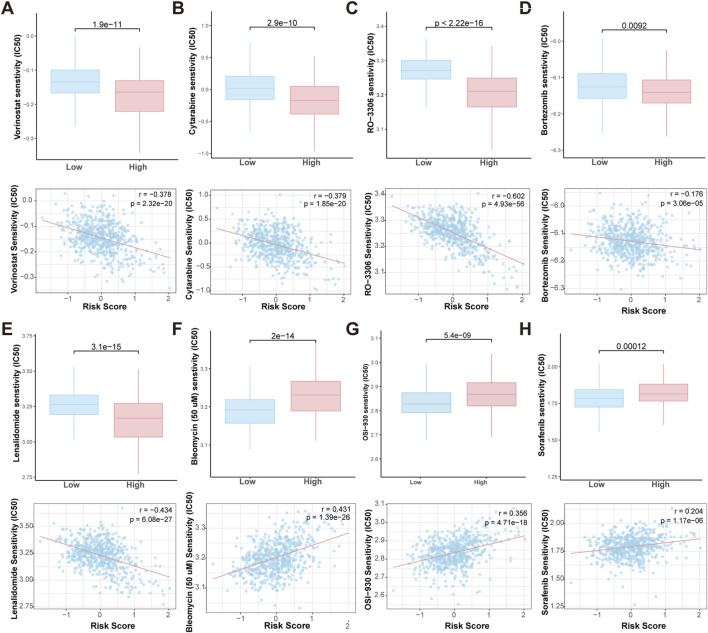
Prediction of drug sensitivity in HMR high-risk and low-risk groups. Boxplot comparing the drug IC50 levels between HMR high-risk and low-risk groups (upper). Scatter plot and correlation analysis demonstrating a correlation between drug sensitivity and risk score (lower). **(A–H)** Vorinostat, Bleomycin, Cytarabine, RO-3306, OSI-930, Sorafenib, Bortezomib and Lenalidomide.

To evaluate the sensitivity of high-risk and low-risk patients to chemotherapeutic agents, we predicted the IC50 values of various chemotherapy drugs and pathway inhibitors using the pRRophetic algorithm. The analysis revealed that high-risk patients were more sensitive to agents like vorinostat (a HDAC inhibitor), cytarabine (a chemotherapy drug commonly used in the treatment of various types of leukemia), RO-3306 (a small molecule CDK1 (Cyclin-Dependent Kinase 1) inhibitor), bortezomib and lenalidomide (targeted therapies used in the treatment MM). Conversely, bleomycin (a chemotherapy drug primarily used in the treatment of certain types of cancer), OSI-930, and sorafenib (VEGFR2 inhibitors) showed higher IC50 values in high-risk patients, suggesting that these patients may resistant to these drugs (Figures 8A–F). These findings suggest that the HMR may could serve as a valuable tool in guiding personalized treatment decisions, helping to optimize chemotherapy drug selection for patients.

## Discussion

In this study, we developed and validated an HMR risk prognostic model based on seven epigenetic regulators using five independent datasets including 2790 MM patients. The HMR risk score was significantly correlated with ISS stage, LDH levels, B2M, ALB, and cytogenetic abnormalities. Multivariate Cox regression analysis confirmed it as an independent prognostic factor for MM. The model effectively stratified patients into high-risk and low-risk groups, with the high-risk group exhibiting significantly worse overall survival. Integration HMR score with clinical parameters improved predictive accuracy and clinical decision benefit. These findings underscore the critical role of epigenetic regulation in MM progression and patient survival.

The seven genes comprising the HMR model (SUZ12, KAT2A, AURKA, BUB1, SUV39H2, UTY, and PCGF5) are critically involved in epigenetic modification, tumor progression, supporting their prognostic value in MM. SUZ12, a core component of the Polycomb repressive complex 2 (PRC2 complex), catalyzes H3K27 trimethylation (H3K27me3) to silence gene expression. SUZ12-mediated H3K27me3 modification inhibits HDAC1 expression, and modulate docetaxel resistance in lung adenocarcinoma to (Jiang et al., 2022a). In T-cell acute lymphoblastic leukemia (T-ALL), SUZ12 mutations activate oncogenic signaling pathways, increasing sensitivity to HDAC6 inhibitors (Broux et al., 2019). High SUZ12 and H3K27me3 expression correlates with poor survival in soft tissue sarcomas (Cho et al., 2018). KAT2A, a histone acetyltransferase, promotes cell proliferation and invasion by acetylating H3K9 and activating E2F1 in colorectal cancer (Han and Chen, 2022). It also enhances resistance to hormone therapy in prostate cancer, further supporting its role in oncogenesis (Lu et al., 2021). Aurora kinase A (AURKA) phosphorylates NSD2 at S56, enhancing its methyltransferase activity and promoting chemotherapy resistance in t(4;14) MM patients (Jiang et al., 2022b). It interacts with BRD4, and targeting both BRD4 and AURKA reduces tumor growth (Xu et al., 2020). AURKA promotes the degradation of tumor suppressor p53, further boosting cell proliferation and oncogenic activity (Park et al., 2018). Furthermore, the AURKA inhibitor AT9283 induced apoptosis and inhibited growth in MM (Santo et al., 2011). BUB1 plays a key role in mitosis by enhancing its kinase activity through TIP60-mediated acetylation, leading to H2A T120 phosphorylation, ensuring accurate chromosome segregation. Non-acetylated BUB1 mutations impair this process, leading to genomic instability (Sun et al., 2024). Additionally, BUB1 promotes GC cell proliferation and metastasis through METTL3-mediated m6A methylation, highlighting its critical role in both mitosis and cancer progression (Wang et al., 2024). SUV39H2 mediates H3K9me3 modification, promoting tumor progression (You et al., 2024), poor survival outcomes (Zheng et al., 2018), and chemoresistance (Vougiouklakis et al., 2018; Wang et al., 2019). UTY (Ubiquitous Transcribed Y), the Y-chromosome paralog of UTX (KDM6A) encode lysine demethylases (KDMs), that catalyzes H3K27me3 demethylation in histone H3 (Black et al., 2012). KDM6A and UTY have significant tumor suppressor activity in several types of cancer (Gozdecka et al., 2018; Shi et al., 2021). PCGF5, a PRC1 component, facilitates Xist-mediated gene silencing through H2A ubiquitination and H3K27 methylation (Almeida et al., 2017). PCGF5 is crucial for neural differentiation in mouse ESCs. Its absence activates the SMAD2/TGF-β pathway, impairing differentiation and weakening H2AK119ub1 and H3K27me3, maintaining gene repression (Yao et al., 2018). Collectively, these genes modulate key histone modifications such as H3K27me3, H3K9me3, H2AK119ub1, and H3K9ac, which play important role in transcriptional regulation, DNA damage response, and chromatin accessibility (Alzrigat et al., 2018). Their dysregulation may disrupt normal gene expression, contributing to increased cell proliferation, impaired apoptosis, and therapeutic resistance, which are features associated with poor prognosis in MM.

Functional enrichment analysis revealed significant upregulation of chromosomal segregation, cell cycle, and proliferation pathways in the high-risk group. These processes promote chromosomal instability (CIN), leading to intratumoral heterogeneity and MM progression (Chen et al., 2025). Furthermore, the high-risk group exhibited a higher TMB, particularly with increased mutations in key oncogenic genes such as KRAS, NRAS, TP53, and MYC. Mutations of the RAS/mitogen-activated protein kinase (MAPK) pathway, including NRAS, KRAS, or BRAF, are found in up to 50% of newly diagnosed MM patients (Walker et al., 2015), and contribute to proteasome inhibitor (PI) resistance (Shirazi et al., 2020). TP53 mutation lead to decreased expression of p53 and associated with poor outcomes and drug resistance in MM (Jovanović et al., 2018). These findings suggest that epigenetic dysregulation may promote genomic instability and disease progression in MM.

Immune infiltration analysis showed reduced neutrophils in the high-risk group, potentially impairing anti-tumor immunity (Chan et al., 2023; Zhu et al., 2024). In parallel, gene expression profiling showed that neutrophil-related genes, including LTF, LCN2, CTSG, ITGAM, and PADI2, were consistently upregulated in the low-risk group, suggesting an enrichment of neutrophil-mediated innate immune activity in these patients. In contrast, the high-risk group demonstrated elevated expression of inflammatory and immunomodulatory genes, such as CXCL9, CXCL10, SOCS1, and IL10RB. These immune signatures may help explain the differential prognosis between groups and may guide personalized immunotherapy approches based on HMR score.

Furthermore, drug sensitivity analysis highlighted the potential of the HMR risk score to guide personalized treatment strategies. High-risk patients exhibited increased sensitivity to HDAC inhibitors (e.g., vorinostat), CDK1 inhibitors, proteasome inhibitors (e.g., bortezomib), and immunomodulatory agents (e.g., lenalidomide), while showing resistance to certain chemotherapy drugs and kinase inhibitors. This highlights the potential for personalized treatment strategies based on HMR risk scores. A recent study showed that AZD1775, a Wee1 inhibitor targeting the cell cycle checkpoint, synergizes with the HDAC inhibitor Vorinostat to induce DNA damage and apoptosis in leukemia cells, including p53-wild type and deficient AML (Zhou et al., 2015). Targeting both epigenetic modifiers (e.g., HDAC inhibitors) and cell cycle regulators (e.g., CDK1 inhibitors) may have synergistic effects in high-risk MM patients.

However, several limitations in this study should be acknowledged. First, although the model was validated by three independent datasets, further validation in prospective clinical cohorts is needed. Second, while this study relied on transcriptomic data, the lack of matched epigenomic datasets limited our ability to link gene expression with chromatin regulatory mechanisms. Integration of multi-omics data in future studies will provide a more comprehensive understanding of the model’s biological basis. Third, functional characterization of the seven genes remains to be fully elucidated. Future studies employing *in vitro* and *in vivo* experiments are needed to elucidate the mechanistic role of these genes in MM pathogenesis and disease progression.

## Conclusion

In conclusion, our study developed and validated a novel HMR risk signature for MM, which exhibited independent prognostic value across multiple cohorts. Integration the HMR score with clinical parameters improved predictive accuracy and clinical decision benefit in both training and validation cohorts. Additionally, the HMR score showed associations with genomic instability, the tumor immune microenvironment, and drug sensitivity, suggesting its potential relevance to MM biology. These findings may contribute to a better understanding of MM progression and offer insights that could inform personalized therapeutic strategies.

## Data Availability

The datasets used and analysed during the current study were obtained from the GEO database (http://www.ncbi. nlm.nih.gov/geo/) and the MMRF-CoMMpass project on the Genomic Data Commons Data Portal (https://portal.gdc.cancer.gov/).

## Supplementary material

SUPPLEMENTARY FIGURE S1
**(A–G)** Kaplan-Meier (KM) curves compare survival rates between the high-expression and low-expression groups, which were defined based on the median expression level of AURKA, BUB1, KAT2A, PCGF5, SUV39H2, SUZ12 and UTY. (H-N) Violin plots illustrating the relationship between HMR risk score and age, gender, lactate dehydrogenase (LDH), β2-microglobulin (B2M), albumin (ALB), cytogenetic abnormalities (Cyto Abn), and ISS stage.

SUPPLEMENTARY FIGURE S2
**(A)** Pairwise correlation analysis among the seven genes. **(B)** Bar plot showing the variance inflation factor (VIF) values for each gene. **(C)** Bar plot showing the coefficients for each gene obtained from multivariate Cox regression.

SUPPLEMENTARY FIGURE S3
**(A)** Univariate and **(B)** multivariate Cox regression analyses identifying prognostic variables in both the training (GSE24080) and validation cohorts (GSE136337 and MMRF-CoMMpass).

## Glossary

MM: Multiple myeloma
HMR: histone modification-related
GEO: Gene Expression Omnibus
TCGA: The Cancer Genome Atlas
LASSO: least absolute shrinkage and selection operator
ISS: International Staging System
HDACs: histone deacetylases
HMTs: histone methyltransferases
FGFR3: fibroblast growth factor receptor 3
MMSET: multiple myeloma SET domain
NSD: Nuclear receptor-binding SET domain
PRC2: Polycomb repressive complex 2
WBM: whole bone marrow
KM: Kaplan–Meier
ROC: receiver operating characteristic
DCA: decision curve analysis
H3K27me3: H3K27 trimethylation
T-ALL: T-cell acute lymphoblastic leukemia
DEGs: Differential expression genes
GO: Gene Ontology
BP: biological processes
CC: cellular component
MF: molecular function
TMB: tumor mutation burden
KEGG: Kyoto Encyclopedia of Genes and Genomes
GSEA: Gene Set Enrichment Analysis
B2M: β2-microglobulin
LDH: lactate dehydrogenase
ALB: albumin
HGB: Hemoglobin
Cyto Abn: cytogenetic abnormalities
CDK1: Cyclin-Dependent Kinase 1
AURKA: Aurora kinase A
γ-H2AX: phosphorylated H2AX
UTY: Ubiquitous Transcribed Y
CIN: chromosomal instability
