# Supplementary material for: Identification and validation of a histone modification-related gene signature to predict the prognosis of multiple myeloma
Source: Front Genet. 2025 Aug 28;16:1613631. doi: 10.3389/fgene.2025.1613631 (PMC12422906; doi:10.3389/fgene.2025.1613631)
Supplement: Supplementary file 1 [file Table1.docx]

| **Data Source** | **Name** | **Patients selected in this study** | **Patient type** | **Biospecimen** | **Applications in this study** |
| --- | --- | --- | --- | --- | --- |
| GEO | GSE24080 | 556 | NDMM | BM CD138+ cell | Training dataset |
| GEO | GSE136337 | 424 | NDMM | BM CD138+ cell | Validation dataset |
| GEO | GSE2658 | 555 | NDMM | BM CD138+ cell | Validation dataset |
| NCI GDC | MMRF-CoMMpass | 853 | NDMM | BM CD138+ cell | Validation dataset  Somatic mutation analysis |
| GEO | GSE136324 | 402 | NDMM | WBM | Immune infiltration analysis. Use 402 patients with paried CD138+ cell expression data |

**Table S1. Datasets used in this study**
