# Supplementary figures and images for: Identification and validation of a histone modification-related gene signature to predict the prognosis of multiple myeloma
Source: Front Genet. 2025 Aug 28;16:1613631. doi: 10.3389/fgene.2025.1613631 (PMC12422906; doi:10.3389/fgene.2025.1613631)

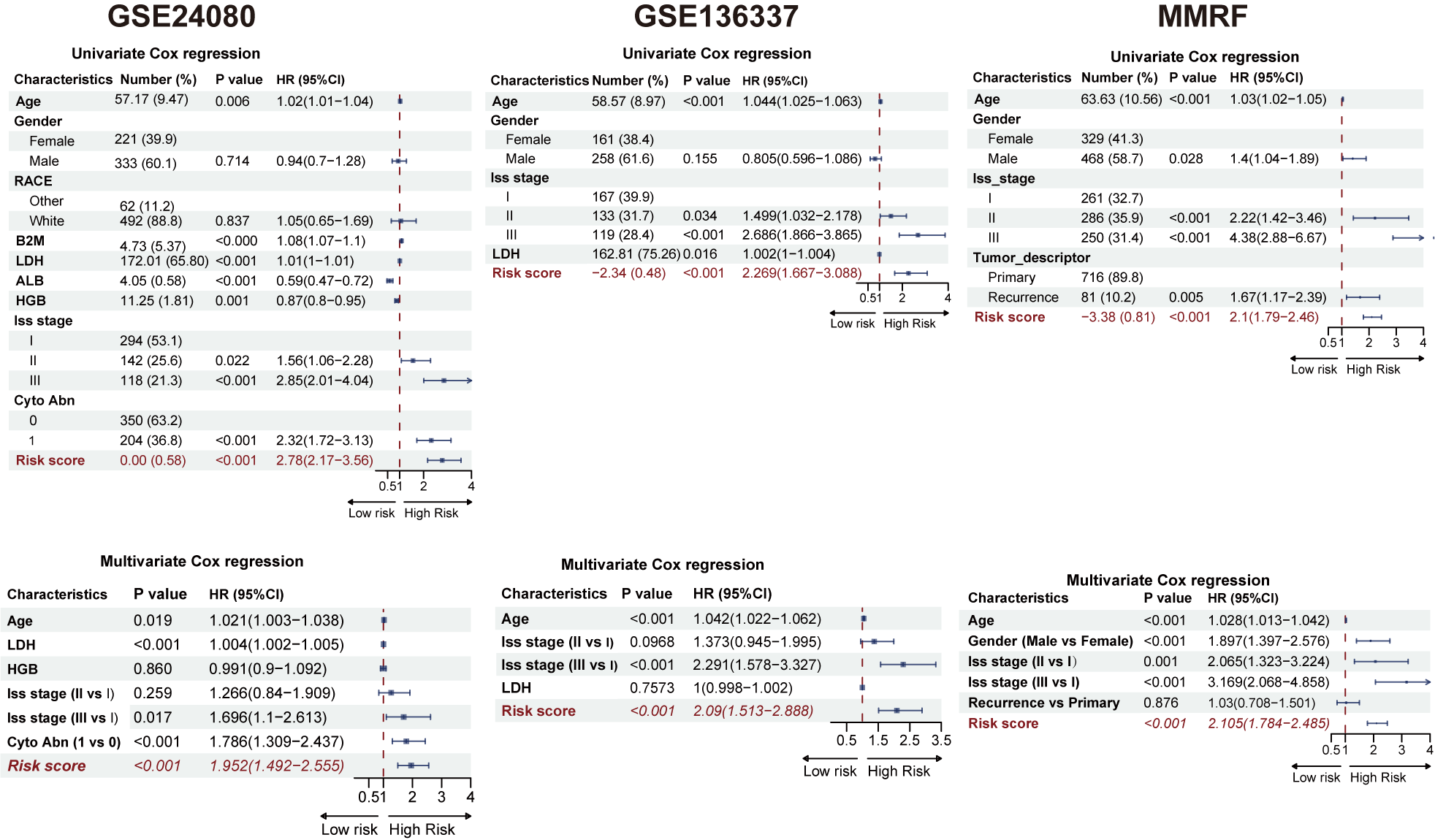

Supplement: Supplementary file 2 [file Image3.tif]

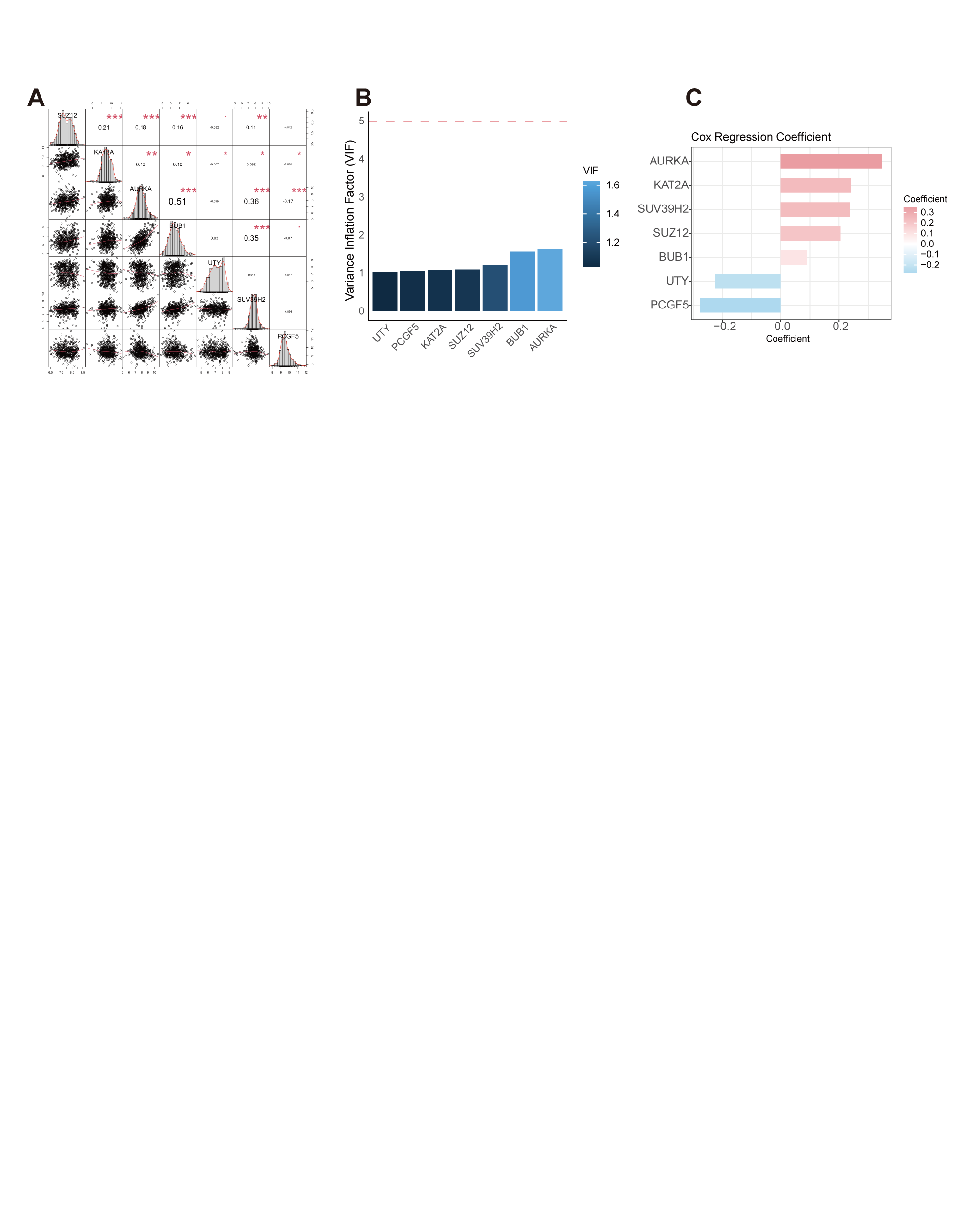

Supplement: Supplementary file 3 [file Image2.tif]

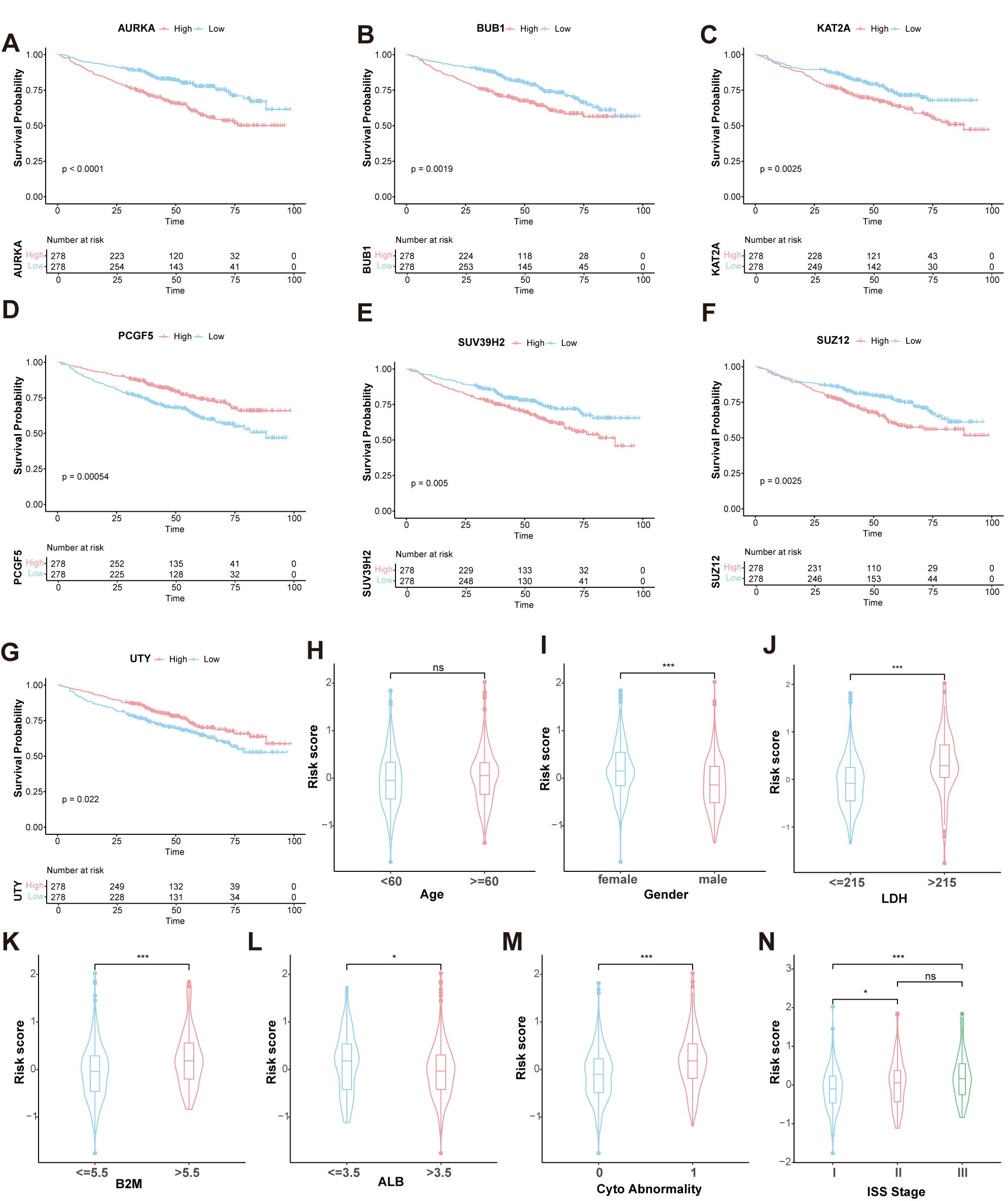

Supplement: Supplementary file 4 [file Image1.tif]
