# Supplementary material for: Identification and validation of a histone modification-related gene signature to predict the prognosis of multiple myeloma
Source: Front Genet. 2025 Aug 28;16:1613631. doi: 10.3389/fgene.2025.1613631 (PMC12422906; doi:10.3389/fgene.2025.1613631)
Supplement: Supplementary file 5 [file Table2.docx]

**Table S2** Comparison of clinical characteristics between HMR high-risk and low-risk groups in the GSE24080 training cohort.

| Variable | Level | High risk (163) | Low risk (391) | P value |
| --- | --- | --- | --- | --- |
| Age (mean (SD)) |  | 57.45 (9.25) | 57.06 (9.58) | 0.659 |
| Gender (%) | Female | 86 (52.8%) | 135 (34.5%) | <0.001 |
|  | Male | 77 (47.2%) | 256 (65.5%) |  |
| Race (%) | Other | 16 (9.8%) | 46 (11.8%) | 0.606 |
|  | White | 147 (90.2%) | 345 (88.2%) |  |
| B2M (mean (SD)) |  | 6.13 (7.39) | 4.14 (4.14) | <0.001 |
| LDH (mean (SD)) |  | 198.88 (85.18) | 160.81 (51.95) | <0.001 |
| ALB (mean (SD)) |  | 3.91 (0.67) | 4.11 (0.53) | <0.001 |
| HGB (mean (SD)) |  | 10.83 (1.70) | 11.43 (1.83) | <0.001 |
| Cyto Abn (%) | 0 | 74 (45.4%) | 276 (70.6%) | <0.001 |
|  | 1 | 89 (54.6%) | 115 (29.4%) |  |
| ISS stage (%) | I | 67 (41.1%) | 227 (58.1%) | 0.001 |
|  | II | 48 (29.4%) | 94 (24.0%) |  |
|  | III | 48 (29.4%) | 70 (17.9%) |  |
| Risk score (mean (SD)) |  | 0.67 (0.38) | -0.28 (0.38) | <0.001 |

**Abbreviation**s: β2-microglobulin (B2M), Lactate dehydrogenase (LDH), Albumin (ALB), Hemoglobin (HGB), Cytogenetic abnormalities (Cyto Abn), International Staging System stage (ISS) stage.
